# Supplementary material for: Variations in Structure among Androecia and Floral Nectaries in the Inverted Repeat-Lacking Clade (Leguminosae: Papilionoideae)
Source: Plants (Basel). 2022 Feb 27;11(5):649. doi: 10.3390/plants11050649 (PMC8912580; doi:10.3390/plants11050649)
Supplement: Supplementary file 1 [file plants-11-00649-s001.zip › Supplement File S1.pdf]

## SUPPLEMENT 1. SEM IMAGES OF ANDROECIA AND NECTARIES OF STUDIED REPRESENTATIVES OF THE IRLC

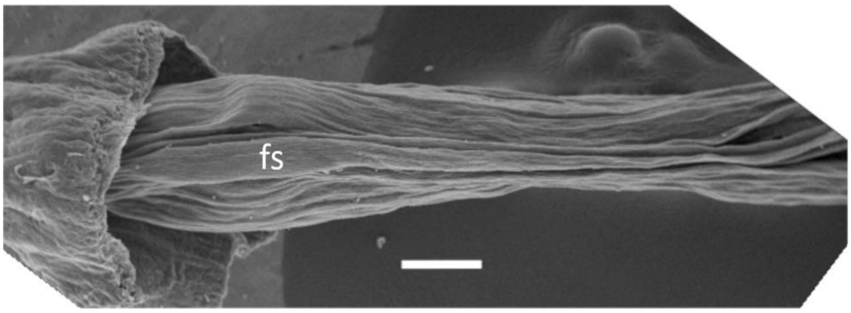


*Alhagi maurorum*, stamens (view from the adaxial side, receptacle is to the left); fs = free stamen. Scale bar: 300 µm.

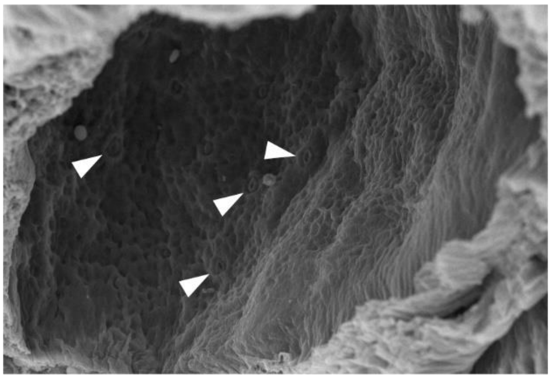

*A. maurorum*, nectariferous stomata (arrowheads). Scale bar: 100 µm.

---

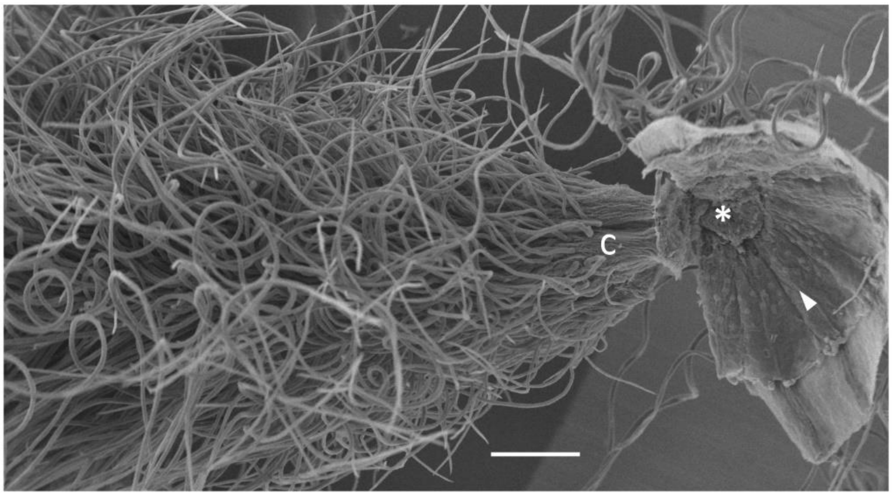


*Astragalus albispinus* (abnormal specimen), base of carpel (c) + portion of receptacle and hypanthium (view from the abaxial side). Asterisk = place where carpel was attached, arrowhead = secretory area on hypanthium. Scale bar: 300 µm.

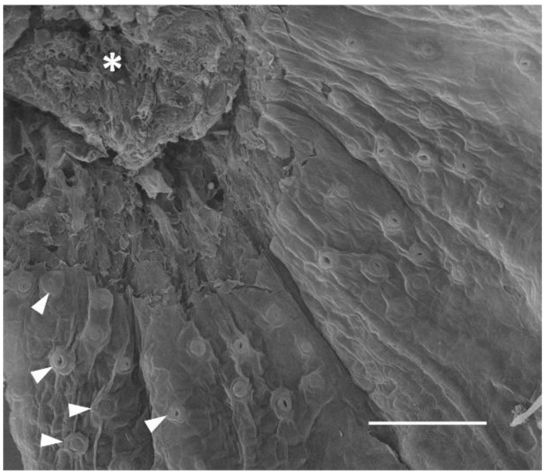


*A. albispinus* (abnormal specimen), nectariferous stomata on receptacle and hypanthium (arrowheads). Asterisk = place where carpel was attached.
Scale bar: 100 µm.

---

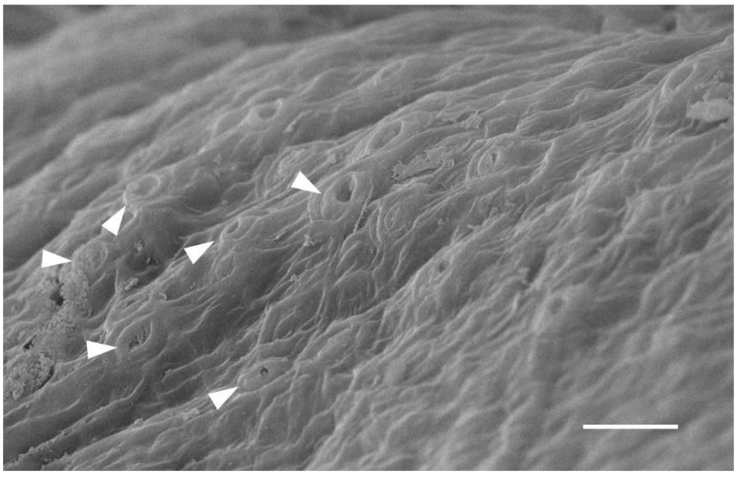

*Astragalus caspicus*, nectariferous stomata on receptacle and hypanthium (arrowheads). Scale bar: 30 µm.

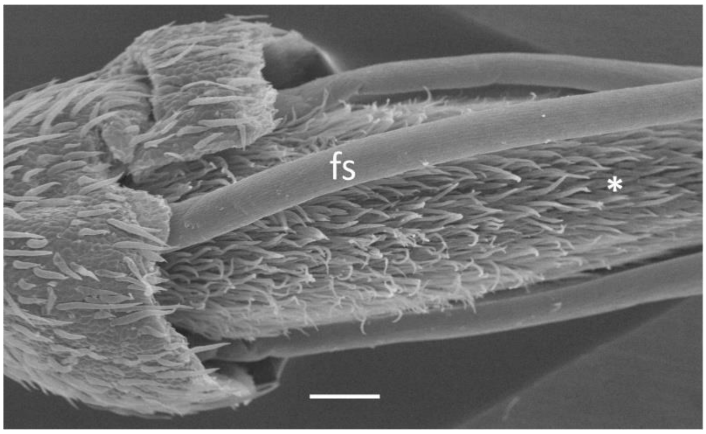


*Astragalus cicer*, stamens (view from the adaxial side, receptacle is to the left). Asterisk = carpel; fs = free stamen. Scale bar: 300 µm.

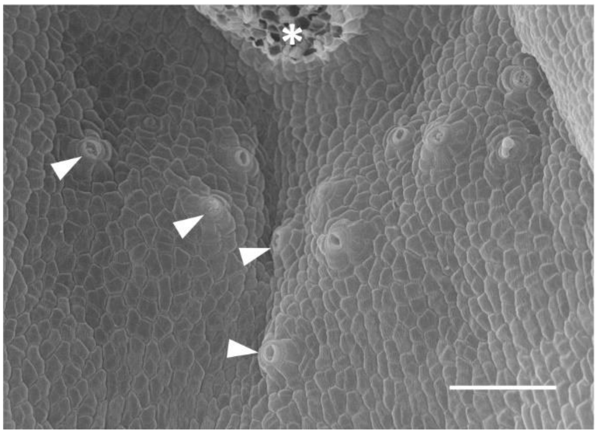

*A. cicer*, abaxial nectariferous stomata (arrowheads). Asterisk = place where carpel was attached. Scale bar: 100 µm.

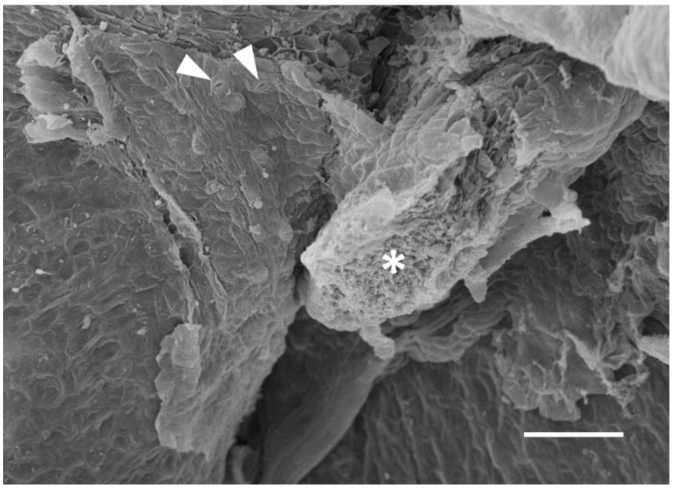

*Astragalus epiglottis*, abaxial nectariferous stomata (arrowheads). Asterisk = place where carpel was attached. Scale bar: 100 µm.

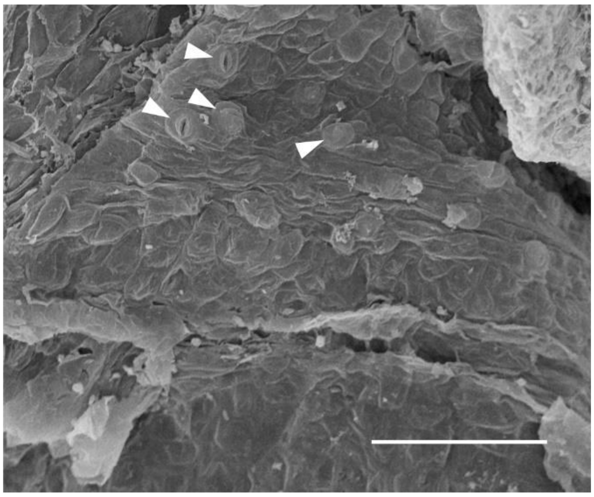

*A. epiglottis*, abaxial nectariferous stomata (arrowheads); enlarged from the image at left. Scale bar: 100 µm.

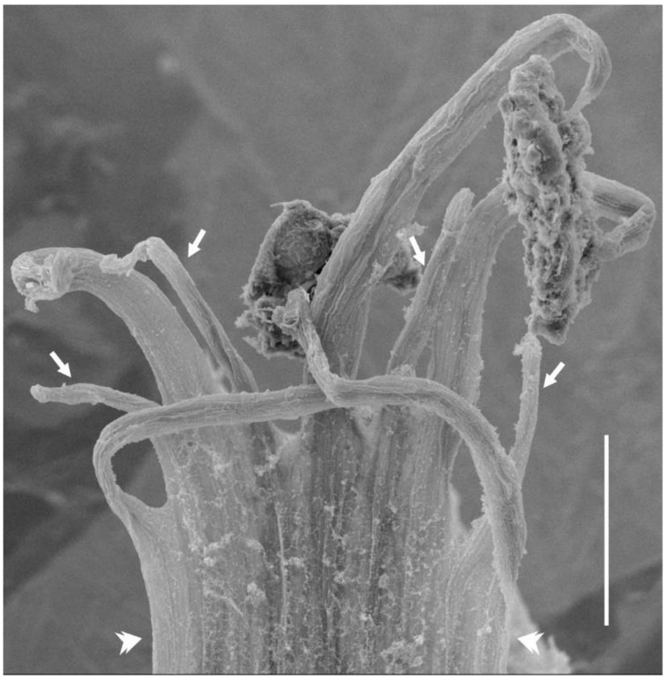

*Astragalus pelecinus*, staminal whorl unfolded (view from the inner side). Arrows = staminodes, double arrowheads = unfused margins of staminal tube. Scale bar: 300 µm.

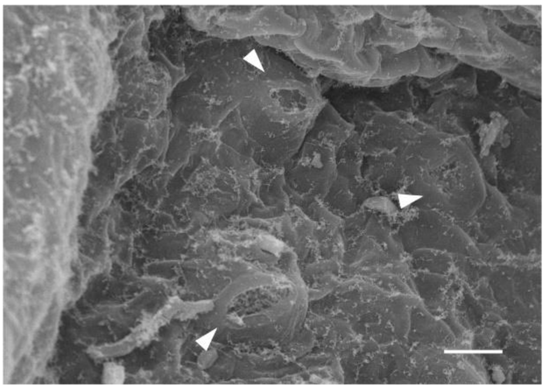

*A. pelecinus*, nectariferous stomata (arrowheads). Scale bar: 10 µm.

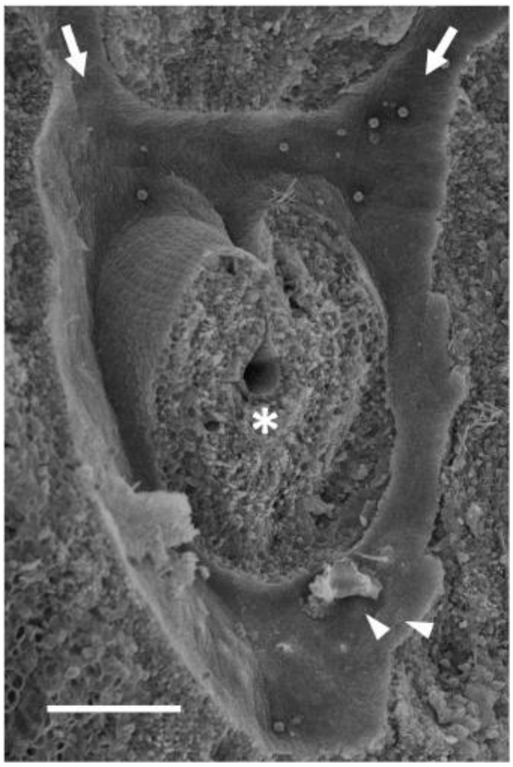



*Caragana arborescens*, abaxial nectariferous stomata (arrowheads). Asterisk = place where carpel was attached; arrows = fenestrae.
Scale bar: 300 µm.

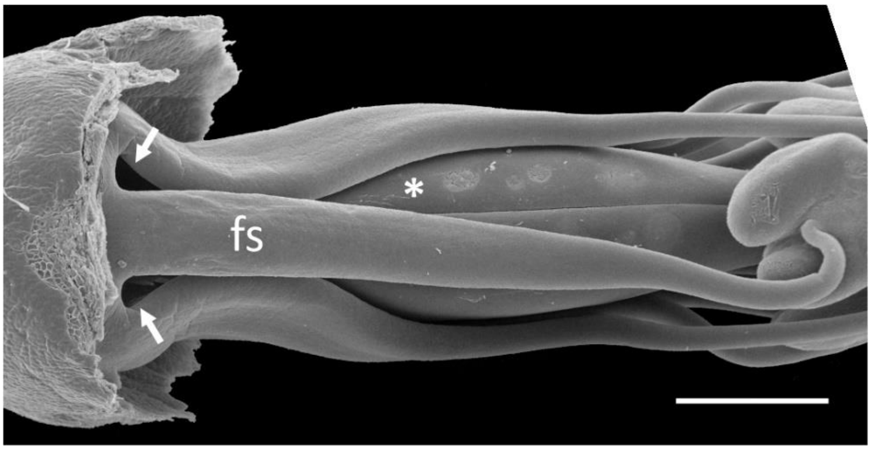


*Colutea arborescens*, stamens (view from the adaxial side, receptacle is to the left). Arrows = fenestrae. Scale bar: 300 µm.

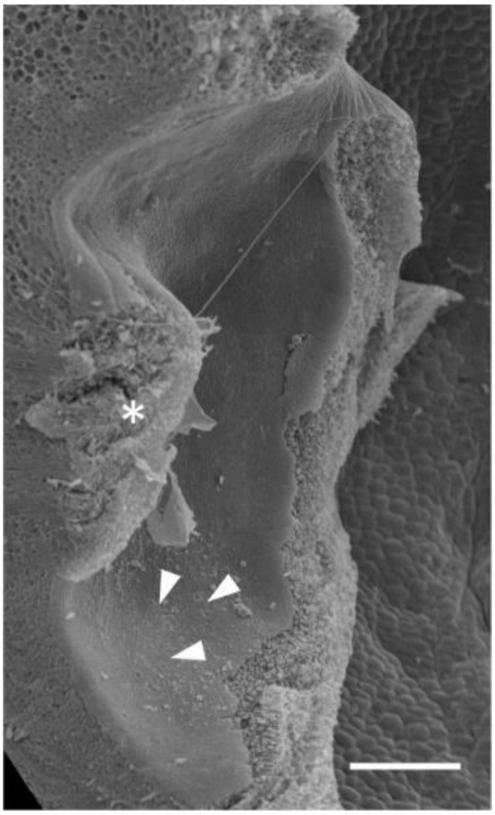


*C. arborescens*, abaxial nectariferous stomata (arrowheads). Asterisk = place where carpel was attached. Scale bar: 300 µm.

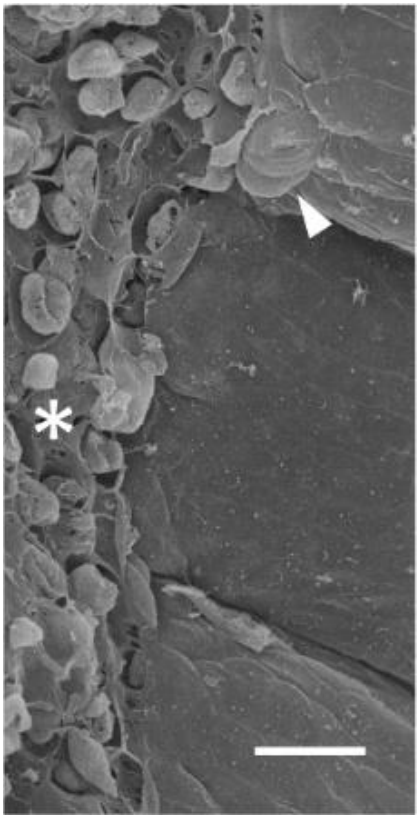

*Galega officinalis*, nectariferous stoma (arrowhead). Asterisk = place where carpel was attached. Scale bar: 300 µm.

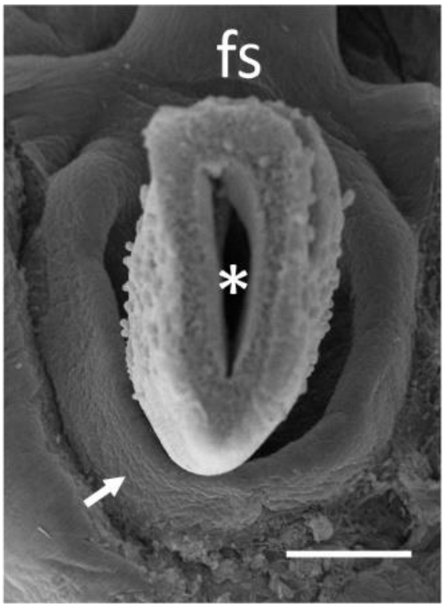


*Lathyrus clymenum*, nectary (arrow). Asterisk = carpel base; fs = free stamen. Scale bar: 300 µm.

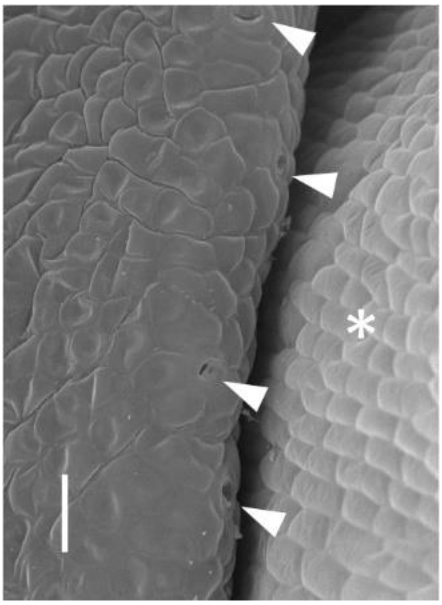

*L. clymenum*, nectary margin with stomata (arrowheads). Asterisk = carpel base. Scale bar: 30 µm.

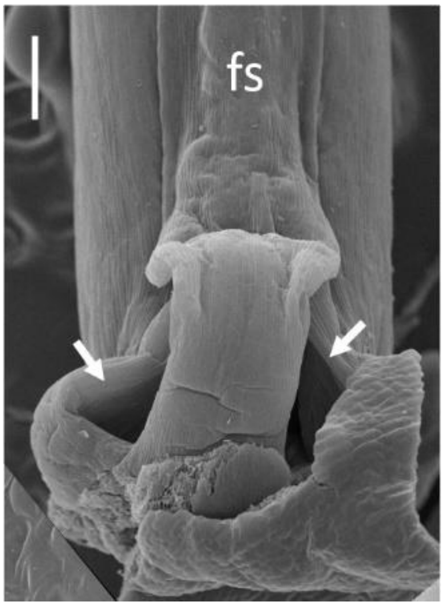


*L. clymenum*, stamens (view from the adaxial side, receptacle is downwards). Arrows = fenestrae; fs = free stamen. Scale bar: 300 µm.

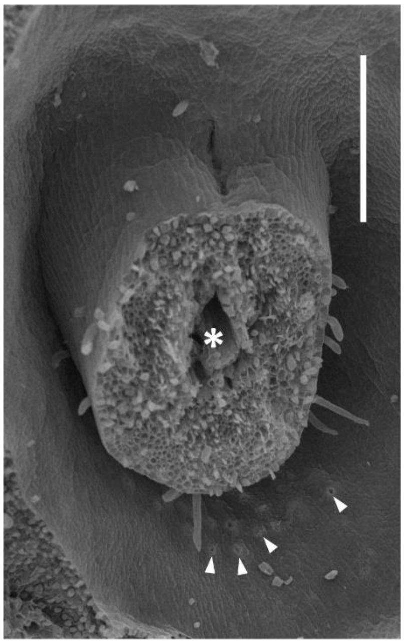

*Lathyrus japonicus* subsp. *maritimus*, abaxial nectariferous stomata on receptacle (arrowheads). Abaxial side is oriented downwards. Asterisk = carpel base. Scale bar: 300 µm.

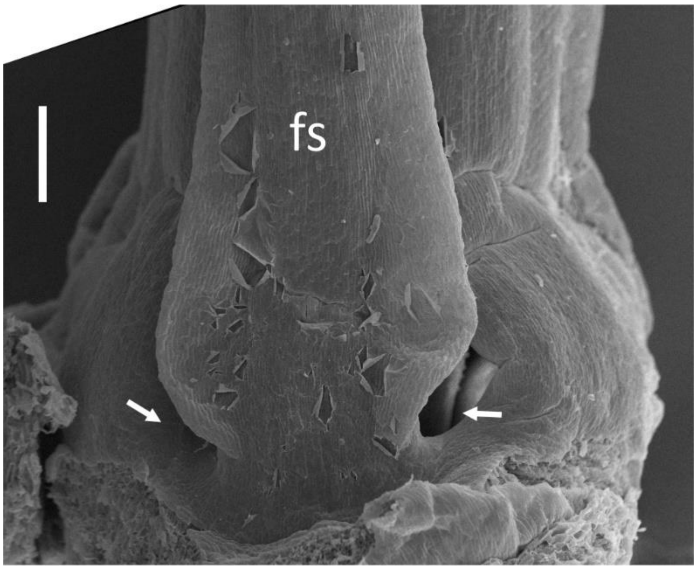


*Lathyrus latifolius*, stamens (view from the adaxial side, receptacle is downwards). Arrows = fenestrae; fs = free stamen. Scale bar: 300 µm.

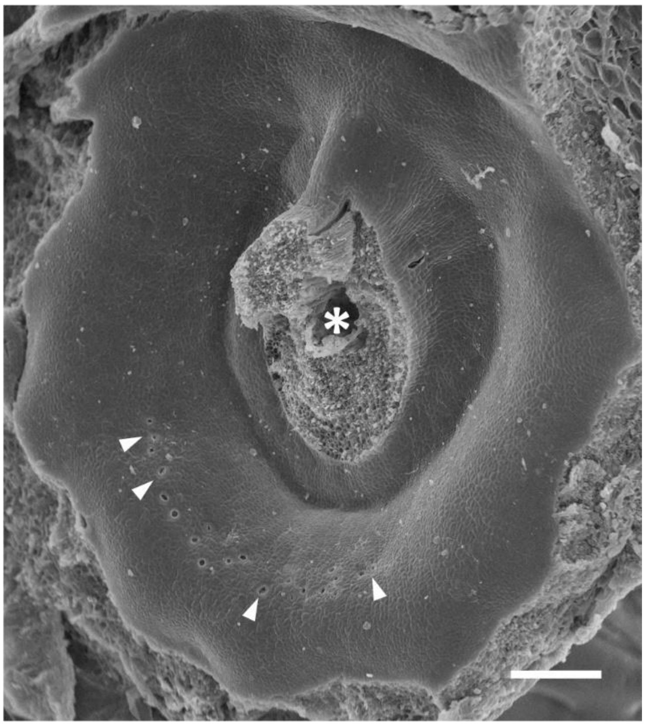

*L. latifolius*, abaxial nectariferous stomata (arrowheads); abaxial side is downwards. Asterisk = place where carpel was attached. Scale bar: 300 µm.

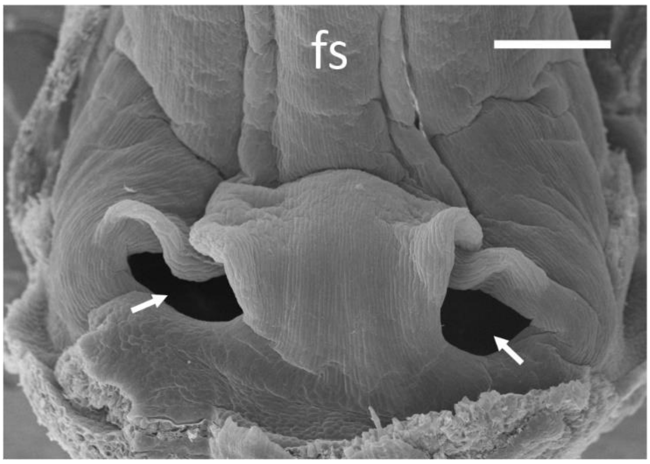


*Lathyrus niger*, stamens (view from the adaxial side, receptacle is downwards). Arrows = fenestrae; fs = free stamen. Scale bar: 300 µm.

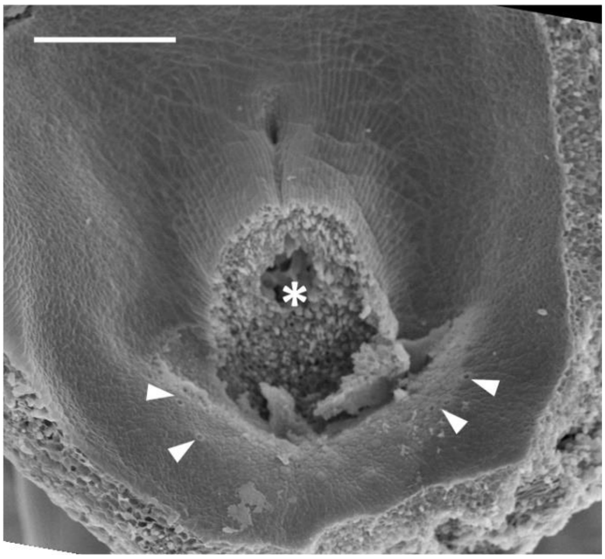

*L. niger*, abaxial nectariferous stomata (arrowheads). Asterisk = place where carpel was attached. Scale bar: 300 µm.

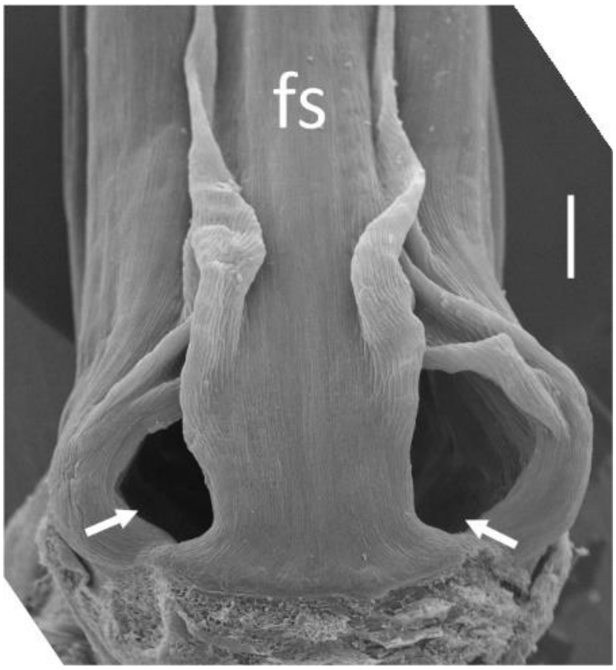


*Lathyrus palustris*, stamens (view from the adaxial side, receptacle is downwards). Arrows = fenestrae; fs = free stamen. Scale bar: 300 µm.

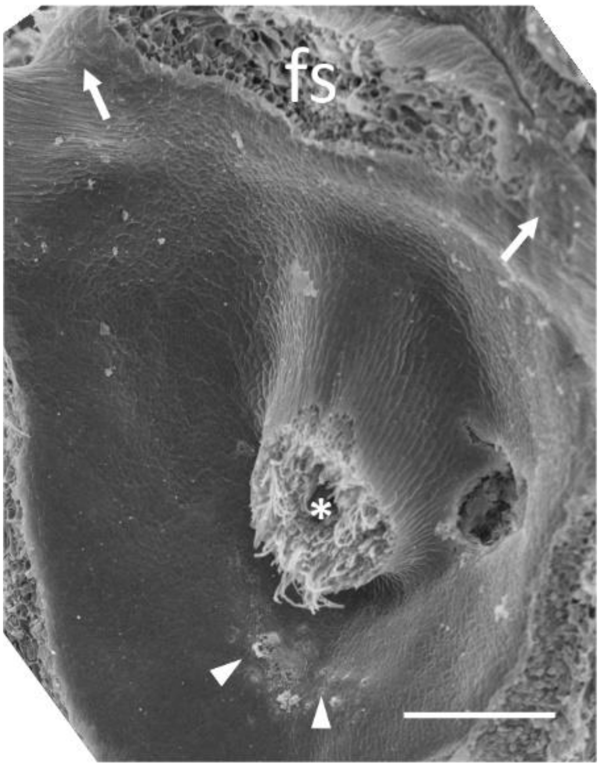


*L. palustris*, abaxial nectariferous stomata (arrowheads; abaxial side is downwards). Asterisk = place where carpel was attached; arrows = fenestrae; fs = former place of free stamen. Scale bar: 300 µm.

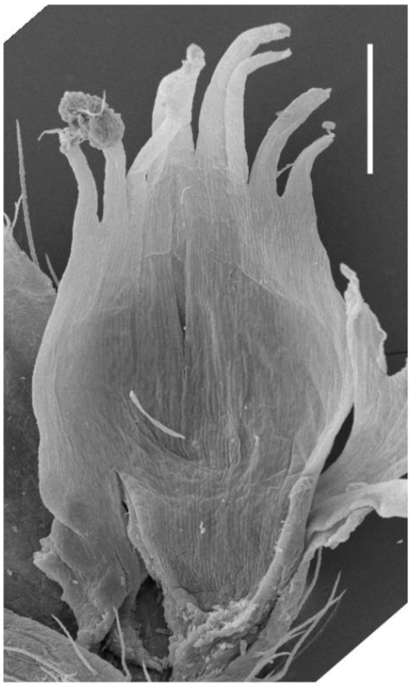

*Medicago lupulina*, unfolded staminal tube (view from inside to the abaxial side, receptacle is downwards). Scale bar: 300 µm.

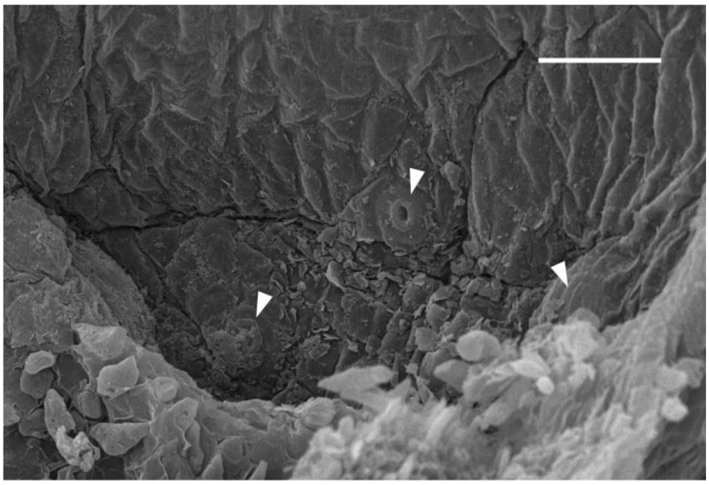

*M. lupulina*, part of receptacle (enlarged from the same specimen as on left image) with nectariferous stomata (arrowheads). Scale bar: 30 µm.

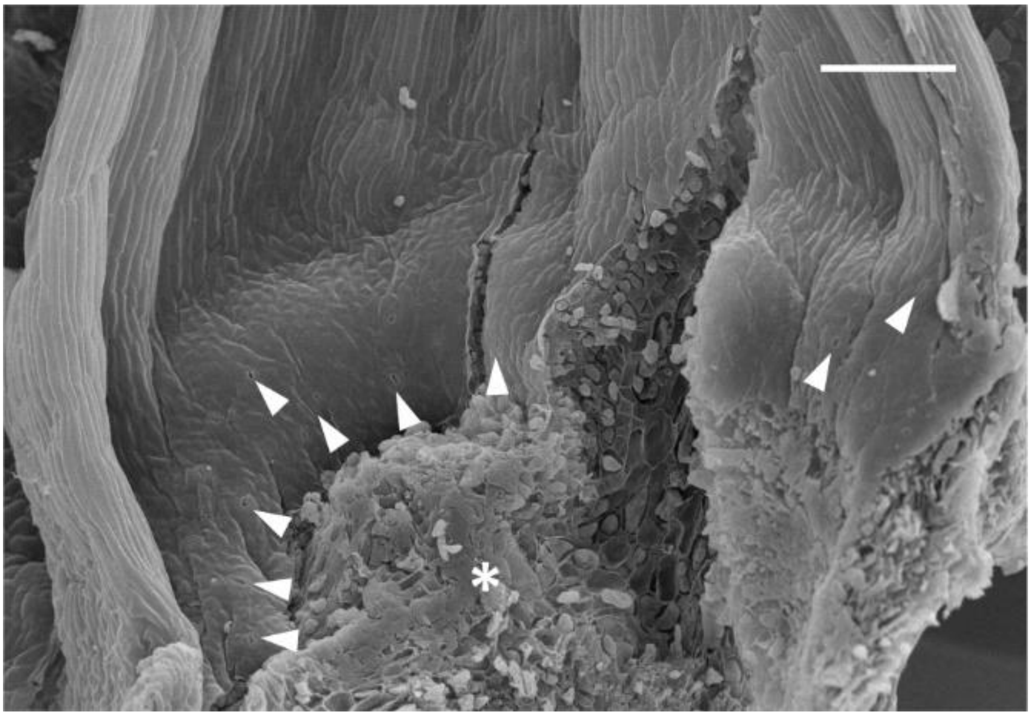

*Melilotus officinalis*, receptacle and hypanthium (view from inside to the abaxial side, receptacle is downwards, abaxial side is upwards) with numerous nectariferous stomata (arrowheads). Asterisk = place where carpel was attached. Scale bar: 100 µm.

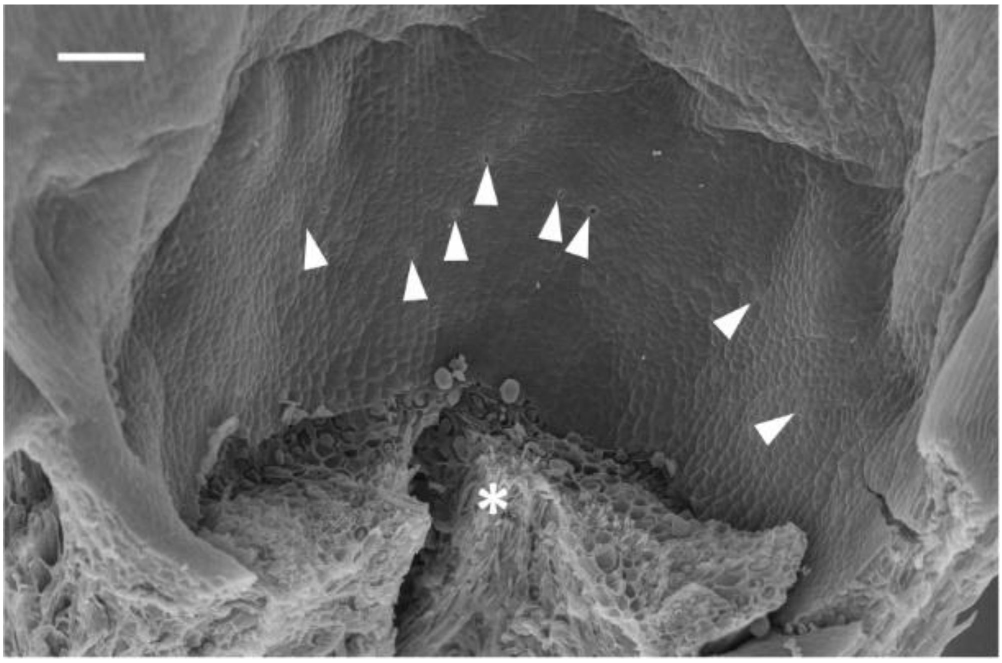


*Onobrychis viciifolia*, receptacle and hypanthium (view from inside to the abaxial side, receptacle is downwards, abaxial side is upwards) with numerous nectariferous stomata (arrowheads). Asterisk = place where carpel was attached. Scale bar: 100 µm.

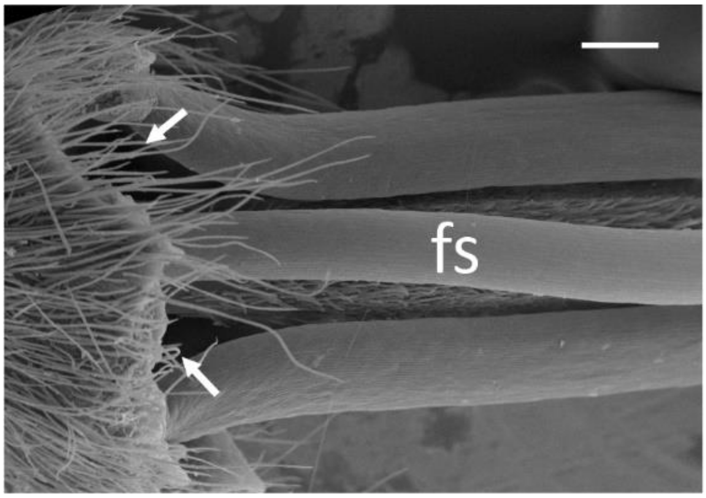


*Oxytropis kamtschatica*, stamens (view from the adaxial side, receptacle is to the left). Arrows = fenestrae, fs = free stamen. Scale bar: 300 µm.

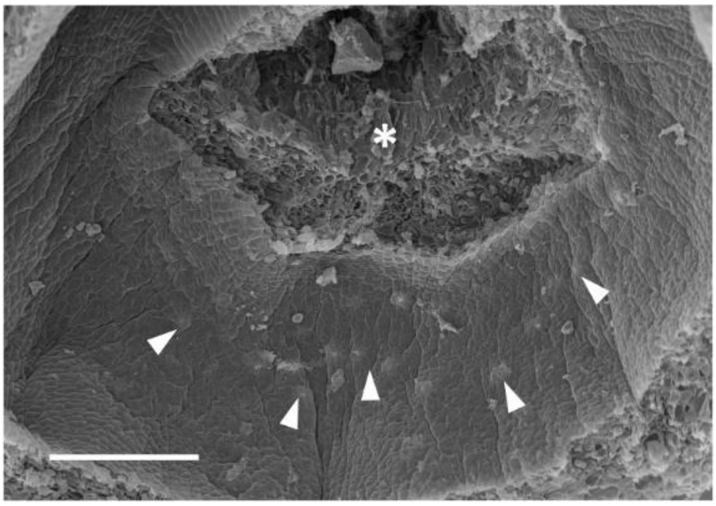


*O. kamtschatica*, abaxial nectariferous stomata (arrowheads; abaxial side is downwards). Asterisk = place where carpel was attached. Scale bar: 300 µm.

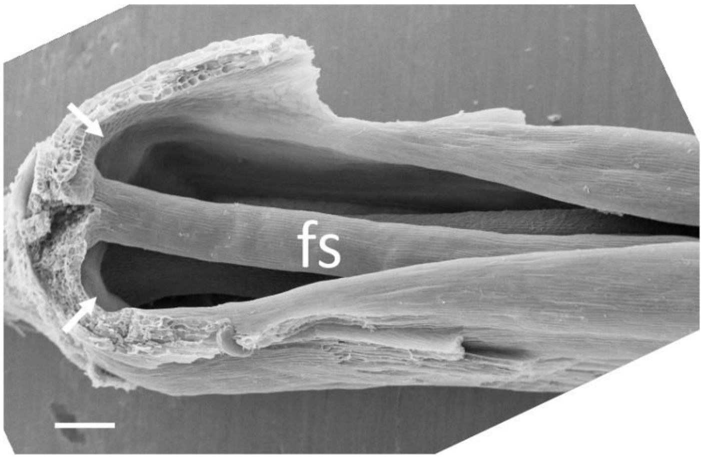


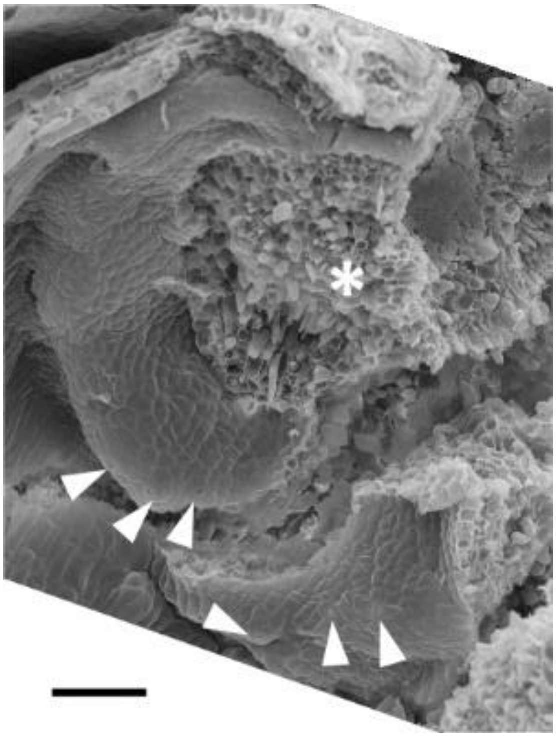

*Trifolium lupinaster*, stamens (view from the adaxial side, receptacle is to the left). Arrows = fenestrae, fs = free stamen. Scale bar: 300 µm.

*T. medium*, nectar disc (partly damaged during dissection) with secretory stomata (arrowheads); abaxial side is downwards. Asterisk = place where carpel was attached. Scale bar: 100 µm.

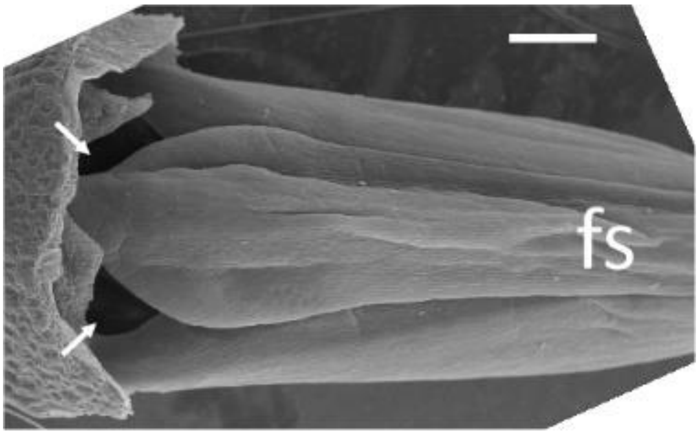


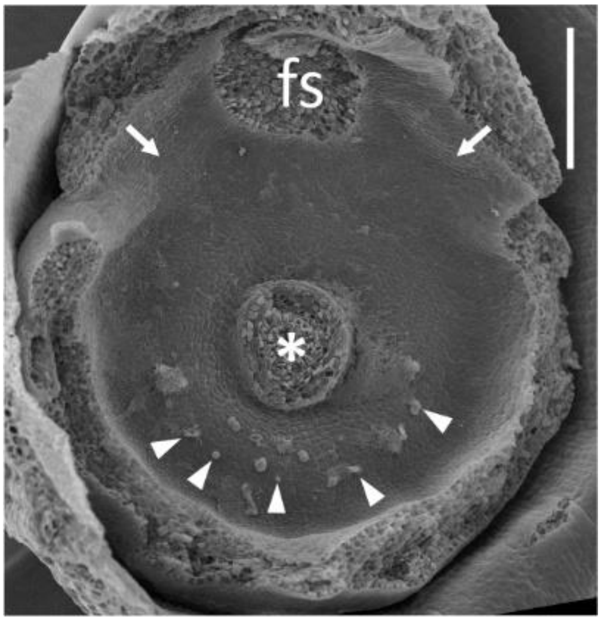


*Trigonella foenum-graecum*, stamens (view from the adaxial side, receptacle is to the left). Arrows = fenestrae; fs = free stamen. Scale bar: 300 µm.

*T. foenum-graecum*, abaxial nectariferous stomata (arrowheads; all are clotted with a secrete), abaxial side is downwards. Asterisk = place where carpel was attached; fs = former place of free stamen; arrows = fenestrae. Scale bar: 300 µm.

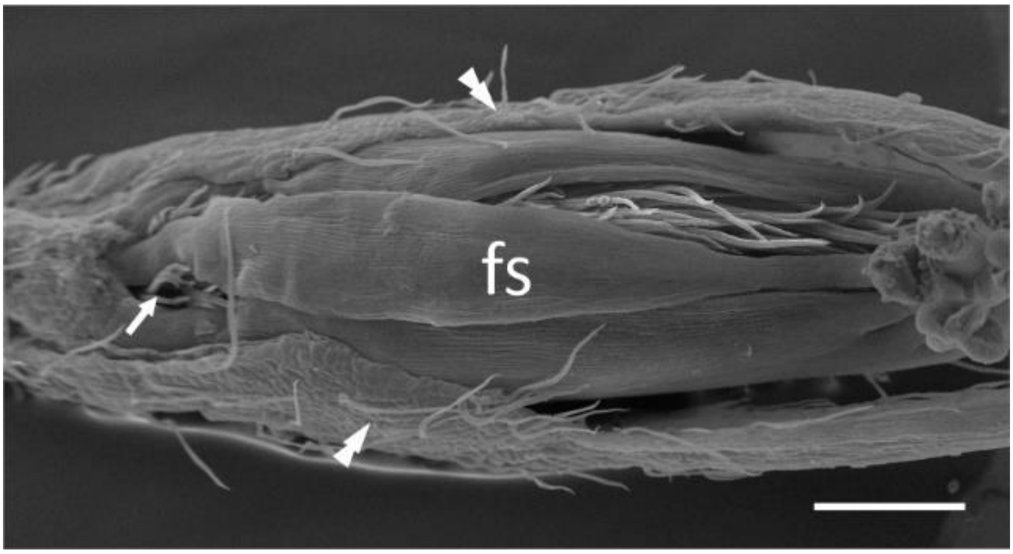


*Vicia hirsuta*, stamens (view from the adaxial side, receptacle is to the left). Arrow = fenestra; double arrowhead = calyx; fs = free stamen. Scale bar: 300 µm.

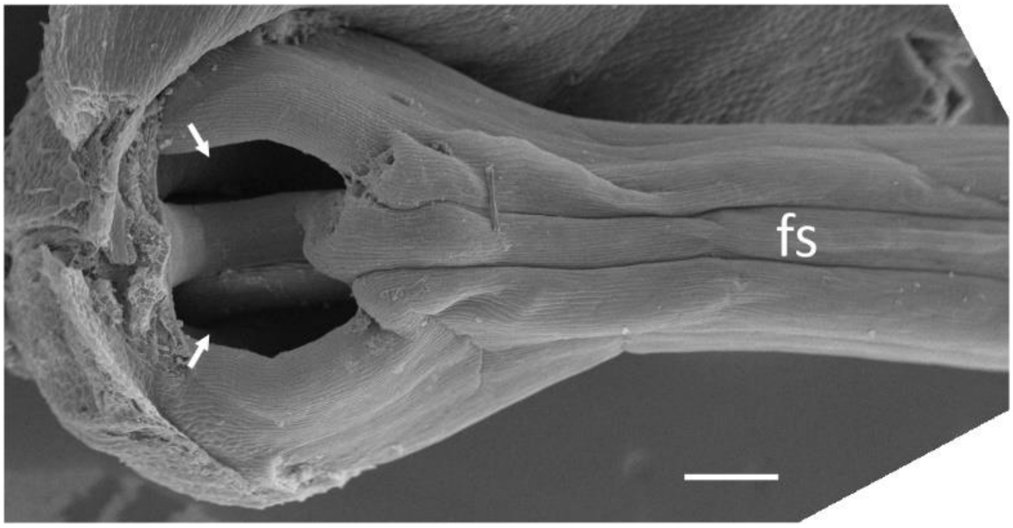


*V. sepium*, stamens (view from the adaxial side, receptacle is to the left). Arrows = fenestrae; fs = free stamen. Scale bar: 300 µm.

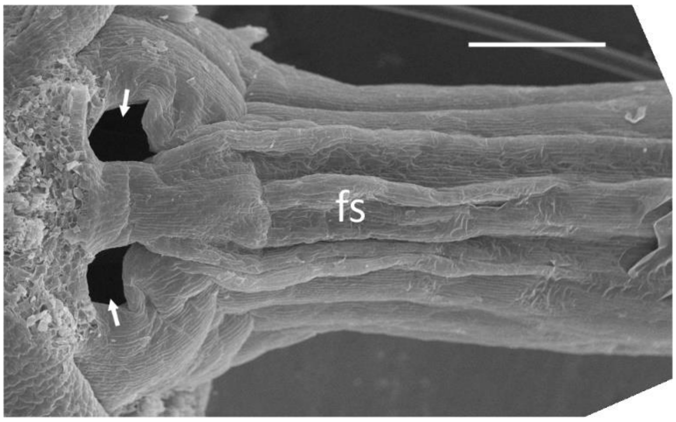


*Vicia sylvatica*, stamens (view from the adaxial side, receptacle is to the left). Arrows = fenestrae; fs = free stamen. Scale bar: 300 µm.

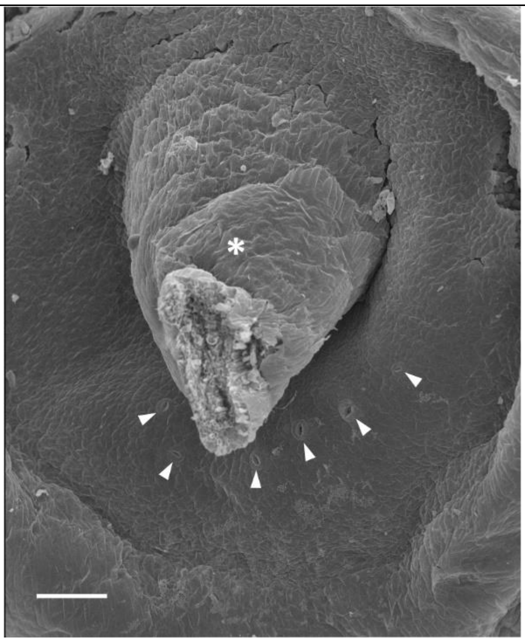


*V. sylvatica*, abaxial nectariferous stomata (arrowheads), abaxial side is downwards. Asterisk = carpel base. Scale bar: 100 µm.

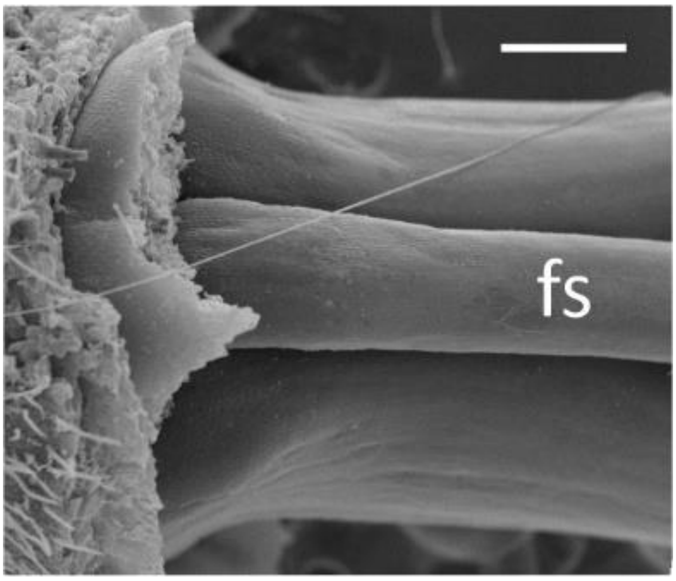


*Wisteria sinensis*, stamens (view from the adaxial side, receptacle is to the left). fs = free stamen. Scale bar: 300 µm.
