# Supplementary material for: Variations in Structure among Androecia and Floral Nectaries in the Inverted Repeat-Lacking Clade (Leguminosae: Papilionoideae)
Source: Plants (Basel). 2022 Feb 27;11(5):649. doi: 10.3390/plants11050649 (PMC8912580; doi:10.3390/plants11050649)
Supplement: Supplementary file 1 [file plants-11-00649-s001.zip › Supplement File S2.pdf]

SUPPLEMENT 2. LIGHT MICROSCOPY IMAGES OF ANDROECIUM IN *GALEGA OFFICINALIS*

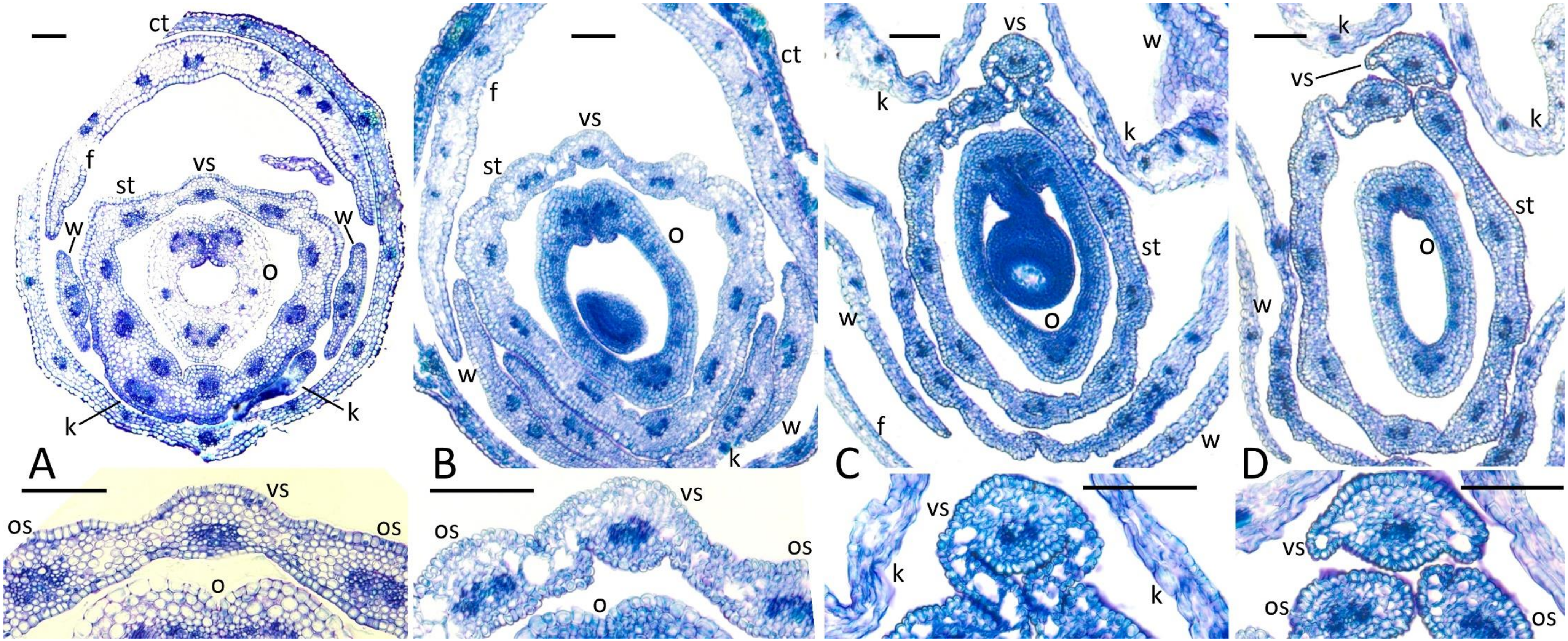

Androecium fusion in *Galega officinalis* as seen in a series of transverse sections of preanthetic flower on different levels (A is the closest to receptacle, while D is the most distal position), light microscopy. On each level, upper image represents overall topography, while lower is a magnification of the vexillary stamen. All images are oriented with their adaxial sides upwards. Key: ct = calyx tube; f = flag; k = keel; o = ovary; os = outer adaxial stamen (as traced by its vascular bundle); s = sepal; st = staminal tube; vs = vexillary stamen (D) or its bundle (A–C); w = wing. Scale bars: 100 µm.
