# Supplementary material for: Variations in Structure among Androecia and Floral Nectaries in the Inverted Repeat-Lacking Clade (Leguminosae: Papilionoideae)
Source: Plants (Basel). 2022 Feb 27;11(5):649. doi: 10.3390/plants11050649 (PMC8912580; doi:10.3390/plants11050649)
Supplement: Supplementary file 1 [file plants-11-00649-s001.zip › Supplement File S3.pdf]

SUPPLEMENT 3. STOMATA ON STAMINAL CONNECTIVES IN SELECTED REPRESENTATIVES OF THE IRLC

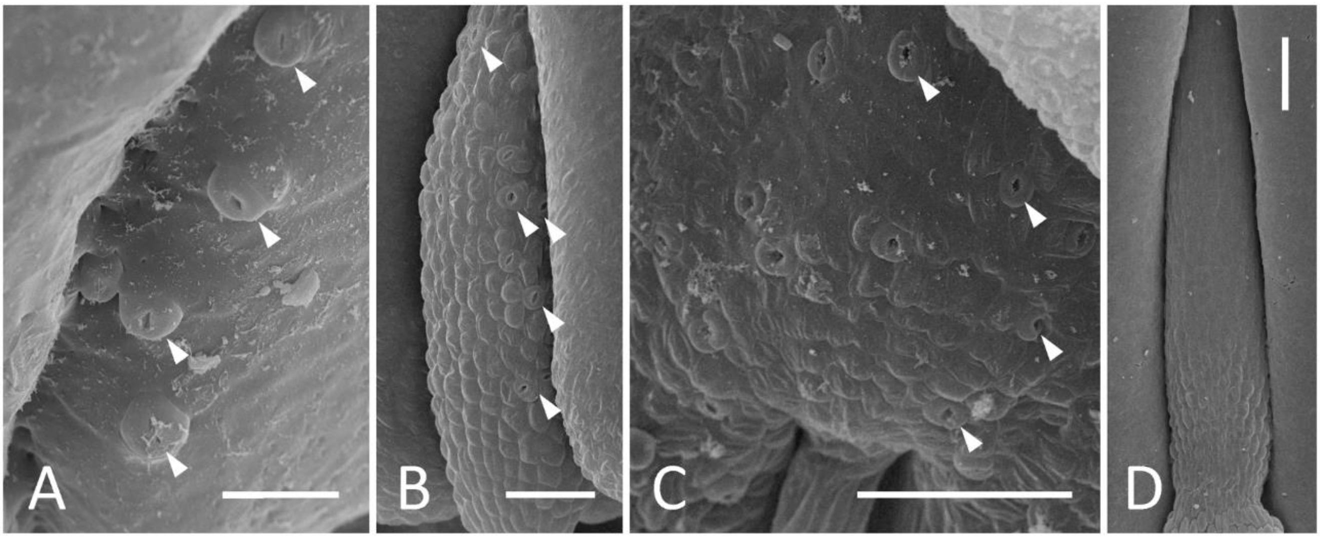

External surfaces of connectives in stamens of selected species. A, *Galega officinalis*; B, *Vicia sepium*; C, *Oxytropis kamtschatica*; D, *Ononis spinosa*. Arrowheads = exemplary stomata. Scale bars: 30 µm (A), 100 µm (B, C, D).
